# Plasma Membranes Modified by Plasma Treatment or Deposition as Solid Electrolytes for Potential Application in Solid Alkaline Fuel Cells

**DOI:** 10.3390/membranes2030529

**Published:** 2012-07-30

**Authors:** Marc Reinholdt, Alina Ilie, Stéphanie Roualdès, Jérémy Frugier, Mauricio Schieda, Christophe Coutanceau, Serguei Martemianov, Valérie Flaud, Eric Beche, Jean Durand

**Affiliations:** 1 Institut Européen des Membranes (UMR 5635-ENSCM, UM2, CNRS), Université Montpellier 2, CC047, Place Eugène Bataillon, Montpellier cedex 5 F-34095, France; Email: marc.reinholdt@univ-poitiers.fr (M.R.); jeremy.frugier@ctisa.fr (J.F.); Mauricio.Schieda@hzg.de (M.S.); jean.durand@iemm.univ-montp2.fr (J.D.); 2 Institut Prime (UPR 3346-CNRS, Université de Poitiers, ENSMA), SP2MI Téléport 2, Boulevard Pierre et Marie Curie, BP 30179, Futuroscope cedex F-86962, France; Email: ilie.alina@yahoo.fr (A.I.); serguei.martemianov@univ-poitiers.fr (S.M.); 3 Institut de Chimie des Milieux et des Matériaux de Poitiers (UMR 7285-CNRS, Université de Poitiers), Université de Poitiers, 40 avenue du Recteur Pineau, Poitiers F-86000, France; Email: christophe.coutanceau@univ-poitiers.fr; 4 Institut Charles Gerhardt (UMR 5253-UM2, ENSCM, CNRS, UM1), Université Montpellier 2, CC1700, Place Eugène Bataillon, Montpellier cedex 5 F-34095, France; Email: valerie.flaud@univ-montp2.fr; 5 Laboratoire Matériaux, Procédés et Energie Solaire (UPR 8521-CNRS, Université de Perpignan), CNRS, Centre Félix Trombe, 7 rue du four solaire, Font-Romeu Odeillo Via F-66120, France; Email: eric.beche@promes.cnrs.fr

**Keywords:** solid alkaline fuel cell, anion exchange membrane, plasma treatment, plasma deposition, glycerol

## Abstract

In the highly competitive market of fuel cells, solid alkaline fuel cells using liquid fuel (such as cheap, non-toxic and non-valorized glycerol) and not requiring noble metal as catalyst seem quite promising. One of the main hurdles for emergence of such a technology is the development of a hydroxide-conducting membrane characterized by both high conductivity and low fuel permeability. Plasma treatments can enable to positively tune the main fuel cell membrane requirements. In this work, commercial ADP-Morgane^®^ fluorinated polymer membranes and a new brand of cross-linked poly(aryl-ether) polymer membranes, named AMELI-32^®^, both containing quaternary ammonium functionalities, have been modified by argon plasma treatment or triallylamine-based plasma deposit. Under the concomitant etching/cross-linking/oxidation effects inherent to the plasma modification, transport properties (ionic exchange capacity, water uptake, ionic conductivity and fuel retention) of membranes have been improved. Consequently, using plasma modified ADP-Morgane^®^ membrane as electrolyte in a solid alkaline fuel cell operating with glycerol as fuel has allowed increasing the maximum power density by a factor 3 when compared to the untreated membrane.

## 1. Introduction

With the growing consumers’ demand for electronic devices, the development of portable power sources, lighter and more compact, is on an ascending curve since the beginning of the last decade [1,2,3,4,5]. Thus, research on the miniaturization of fuel cells, which are among the most attractive power generation systems of the future (due to their high energy converting efficiency and their cleanliness), has become an important challenge. Most of the efforts to prepare devices dedicated to this market of portable and mobile electronic systems are focused on the development of direct methanol fuel cells (DMFC) based on proton exchange membranes (PEM), essentially constituted by perfluorosulfonated polymers like Nafion^®^ [1,5,6]. The high cost of such equipment due to the use of noble metals like Pt at the electrodes and the relatively high price of perfluorosulfonated polymers, the slow reaction kinetics at the anode and the important methanol cross-over through the membrane are the main reasons for their limited industrialization in large scale production [1,3,5,6]. In this context, solid alkaline fuel cells (SAFC), which offer the advantages of using liquid fuel and non-noble metal catalysts, seem to be particularly competitive [7,8,9,10]. Since the membrane is an anion conductor, the SAFC is a hybrid between solid polymer electrolyte fuel cell (SPEFC) and alkaline fuel cell (AFC). The solid nature of the electrolyte in the SAFC is another advantage compared to AFC when considering the alkaline electrolyte carbonation process. 

Among alcohols and polyols as potential fuels for SAFC, glycerol is a by-product from the methanolysis of vegetable oils employed for bio-fuel production; so its use as a fuel in SAFC would bring it some added-value [11,12]. Indeed, when compared to methanol (MeOH), glycerol has the advantage of being a non-toxic and non-industrial chemical. While compared to ethanol (EtOH), glycerol has a relatively good theoretical energy density (5.0 kWh kg^−1^ compared to 8.0 kWh kg^−1^ for EtOH) and a higher recovering yield of the whole theoretical energy (up to 30% and 70% for EtOH and glycerol, respectively). Another recently emerging fuel for SAFC is sodium borohydride (NaBH_4_) [13,14]. The latter has several advantages: NaBH_4_ has a higher energy density than methanol (9.3 and 6.1 kWh kg^−1^ for NaBH_4_ and MeOH, respectively); it is relatively stable in alkaline solutions, can be easily stored and handled, and is non-toxic. Both glycerol and NaBH_4_ will be envisaged as fuels in this study. 

As promising solid electrolyte for SAFC, the ADP-Morgane^® ^membrane has been previously used in DMFC and its ionic conductivity, methanol permeability (both lower than Nafion^®^) and performance when assembled in Membrane Electrode Assembly (MEA) have been measured [15,16]. More recently, the use of ADP-Morgane^®^ membrane in direct borohydride fuel cells (DBFC) has emerged [12,17]. These works have essentially shown that the membrane retention to NaBH_4_ and water (at the anode and cathode interfaces, respectively) had to be improved. Now, surface modification of membranes using plasma processes is a promising strategy for enhancement of liquid or gas retention. This modification may consist of the Plasma Enhanced Chemical Vapor Deposition (PECVD) of an ultra-thin highly cross-linked film at the membrane surface or of the physical cross-linking of the membrane surface under the action of a non-condensable gaseous plasma phase [18,19,20,21,22,23,24,25]. PECVD is an environmental friendly method allowing the deposition of dense and very homogeneous thin films (from a few nanometers to several microns) with a good adhesion on any support [19,20,21,26]. PECVD films are tri-dimensional materials formed by the random recombination of the precursor fragments, due to very complex and non-specific chemical reactions occurring in the plasma glow discharge [27,28]. In spite of their disorganized structure, plasma films can present functional properties; for example, choosing a precursor containing tertiary amines makes possible the preparation of hydroxide conducting plasma films as proven by our group [19,29]. Moreover plasma films are generally very highly cross-linked which gives them high chemical and thermal stabilities [18,19,26,27,30,31]. Due to its intrinsic functional properties and cross-linked character, a PECVD film deposited on the surface of a polymer membrane is likely to positively tune the functional properties of the membrane support. The present work proposes to demonstrate the feasibility to improve the retention of ADP-Morgane^®^ membrane to fuel while maintaining its hydroxide conducting capacity by the addition of an upper plasma film on its surface. Such an approach has not been investigated yet for fuel cell membranes. In parallel cross-linking plasma treatment using non-condensable gaseous phase will also be envisaged with the same objective. The interest of such a cross-linking plasma treatment in term of fuel retention enhancement has recently been proven in the case of proton conducting membranes (solely Nafion^®^) [22,23,24]. The first attempt to modify Nafion^®^ by using argon plasma has been done by Choi *et al.* [22]; they have shown that the plasma process essentially increased the membrane surface roughness and decreased the methanol permeability. Lue *et al.* have observed similar phenomena, though they also observed that the ion exchange capacity and proton conductivity were slightly reduced while water uptake, mechanical strength and thermal stability were not significantly changed [23]. Bae *et al.* have additionally demonstrated (performing FT-IR and XPS analyses) that an etching effect of the plasma could be responsible for proton conductivity decrease due to removal of sulfonic acid groups and break of the ether linkages at the membrane surface [24]. The present work aims at demonstrating the feasibility of simultaneous fuel retention improvement and ion conduction maintenance induced by physical plasma treatment.

In this study, two different kinds of synthetic anionic conducting polymer membranes have been plasma modified and characterized. The first kind is the commercial ADP-Morgane^®^ membrane from Solvay (Belgium), which is a cross-linked post-quaternized ethylene tetrafluoroethylene-chloromethylstyrene copolymer. The second is a membrane recently developed by specific polymers (Montpellier, France), named AMELI-32^®^, which is a cross-linked poly(aryl-ether) polymer containing quaternary ammonium functionalities and which has the advantage of being less expensive than ADP-Morgane^®^, because of its structural nature and chemical composition. Two different plasma modifications have been performed: plasma treatment using argon as gaseous phase (on both ADP-Morgane^®^ and AMELI-32^®^) and plasma deposition using triallylamine as precursor (on ADP-Morgane^®^ only). The main studied plasma parameters have been the discharge power (*P_w_*), and in some reduced extent the duty cycle (*DC*) and the plasma glow duration (*τ*). The different membranes prepared in this project have been characterized by scanning electron microscopy (SEM) and X-ray photoelectron spectroscopy (XPS) in order to collect morphological and structural properties. Additionally, ion exchange capacity (*IEC*), water content, permeability to liquid fuels (NaBH_4_ and glycerol) and hydroxide conductivity (by electrical impedance spectroscopy (EIS)) measurements have been performed and correlated to the plasma parameters and morphological/structural characterizations. Lastly, the performance of plasma-modified ADP-Morgane^®^ as electrolyte has been investigated by conducting fuel cell tests in SAFC using glycerol as fuel and home-made electrodes.

## 2. Results and Discussion

### 2.1. Morphological and Structural Properties

#### 2.1.1. SEM Observations

**Figure 1:**
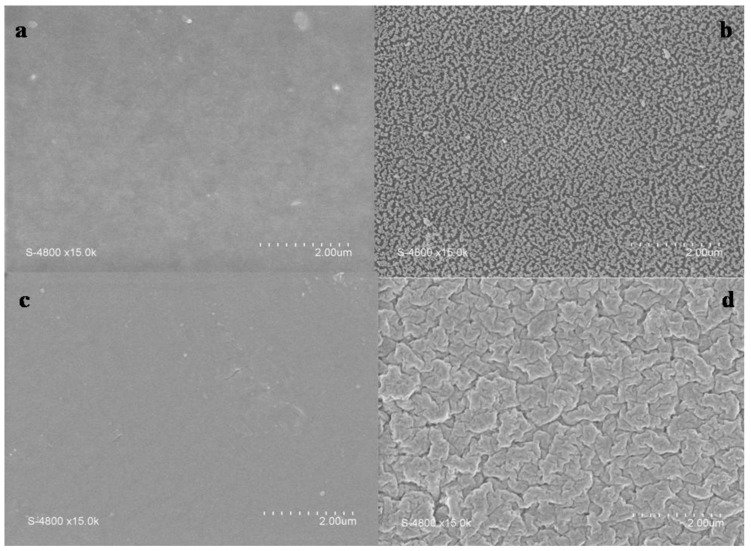
Scanning electron microscopy (SEM) pictures of representative faces of (**a**) pristine ADP-Morgane^®^; (**b**) plasma modified ADP-Morgane^®^ (*P_w_* = 70 W, *DC* = 100% and *τ* = 10 min); (**c**) pristine AMELI-32^®^ and (**d**) plasma modified AMELI-32^®^ (*P_w_* = 60 W, *DC* = 100% and *τ* = 20 min) membranes.

The effect of the argon plasma treatment on membranes morphology may be observed in Figure 1 where surfaces of plasma treated and untreated membranes are compared. One can observe that morphological changes occur for both ADP-Morgane^®^ and AMELI-32^®^ membranes. Whereas treated ADP-Morgane^®^ surfaces show a kind of vermicular morphology, treated AMELI-32^®^ ones show more flake-like morphology. Similar surface modification as the ADP-Morgane^®^ membrane one has been previously observed in the case of Nafion^® ^membrane [24] and has been attributed to the reduction of grain size of the polymer matrix or/and to lower mechanical properties.

**Figure 2:**
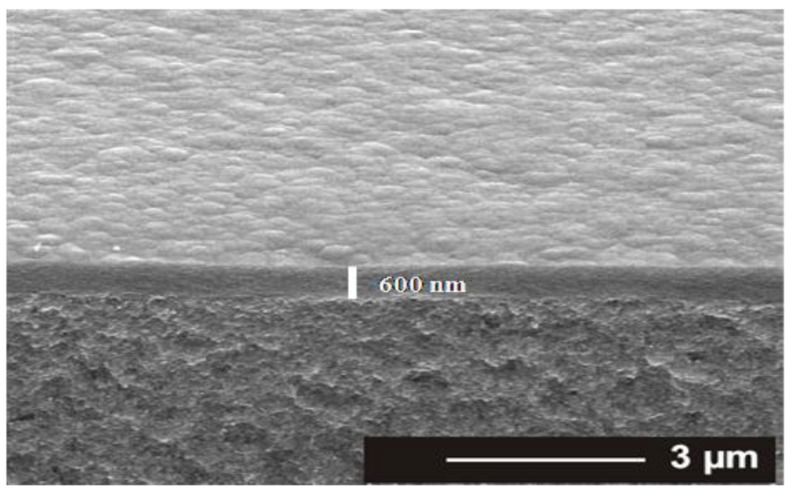
Cross-sectional SEM pictures of a representative plasma deposited TAA polymer thin film on ADP-Morgane^®^ membrane, performed in the following conditions: *P_w_* = 40 W, *DC* = 10% and *τ* = 60 min.

**Figure 3:**
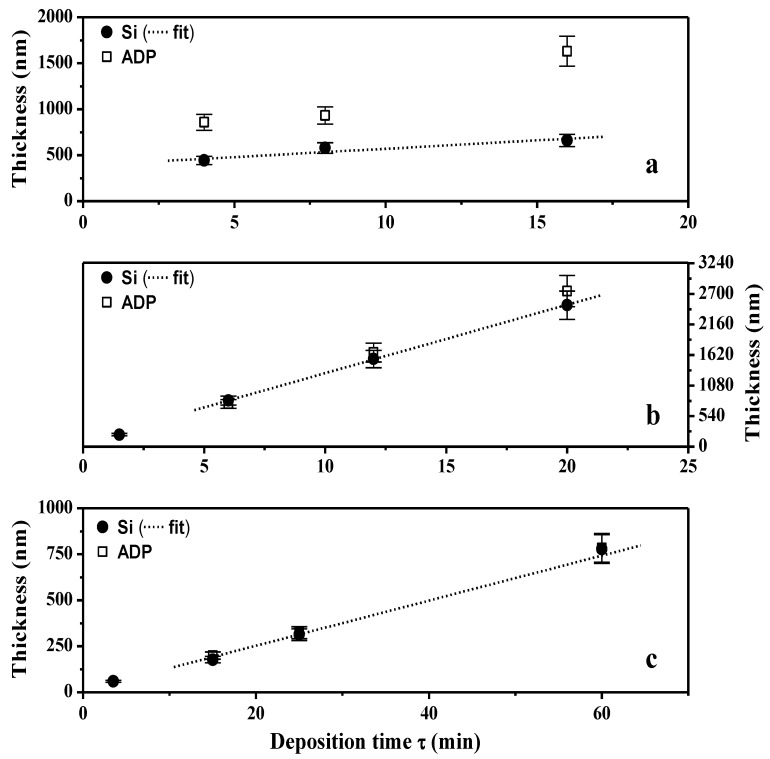
Thin films thickness as a function of deposition time (*τ*) both on silicon wafer and ADP-Morgane^®^ membrane. Plasma polymerization was performed in the following conditions: (**a**) *P_w_* = 150 W, *DC* = 100%; (**b**) *P_w_* = 40 W, *DC* = 100%; (**c**) *P_w_* = 40 W, *DC* = 10%. The straight dotted lines labeled as fits correspond to linear regressions of the deposit growth on silicon wafer for deposition times above 2 min (once the permanent regime reached).

**Figure 4:**
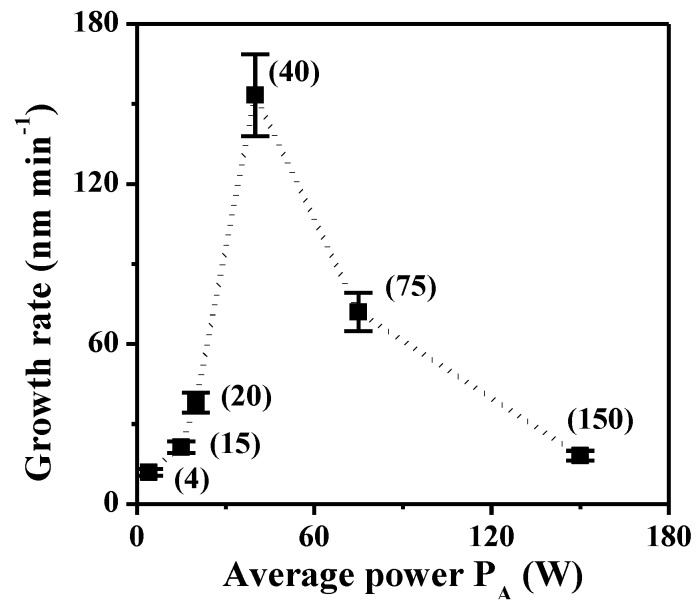
Growth rate of plasma polymers deposited on silicon wafer as a function of average input power (*P_A_*, given in brackets).

The TAA plasma films deposited on silicon wafer and on ADP-Morgane^®^ membrane were also examined using SEM (Figure 2). Whatever the support, all samples exhibit defect-free and homogeneous thin films whose surface is smooth with some shallow waves. The evolution of the film thickness (*Th*) as a function of different synthesis parameters (Figure 3) show that the thickness of films deposited on silicon wafer is increasing linearly with the plasma discharge duration *τ*, as soon as a permanent regime is reached (*τ* > 2 min), regardless of the *P_w_* and *DC* values. The linearity of *Th* for plasma deposits on ADP-Morgane^®^ membrane is not as good as on silicon wafer, certainly due to the roughness of the membrane surface which may induce some film thickness irregularities. Growth rate values can be deduced from linear regressions of *Th* = F(*τ*) curves for *τ* values above 2 min (permanent regime). The curve representing the film growth rate on silicon wafer as a function of the average input power *P_A_* is given in Figure 4. Its profile is characteristic of two different plasma condition regions [31]. The first region (*P_A_* ≤ 40 W here) is known as the energetic deficient region, where an increase of *P_A_* induces an increase of the number of monomer fragments, and consequently a raise of the film growth rate. The second region (*P_A_* > 40 W here) corresponds to the monomer deficient region, in which an increase of *P_A_* leads to more fragmented and so smaller species, reducing the film growth rate and inducing more reticulated and dense polymers. This bimodal evolution is well-known as the competitive ablation and polymerization process (CAP process) [27,30,31]. A similar phenomenon could have been observed for growth rates of films deposited on ADP-Morgane^®^ membrane.

#### 2.1.2. XPS Analyses

**Figure 5:**
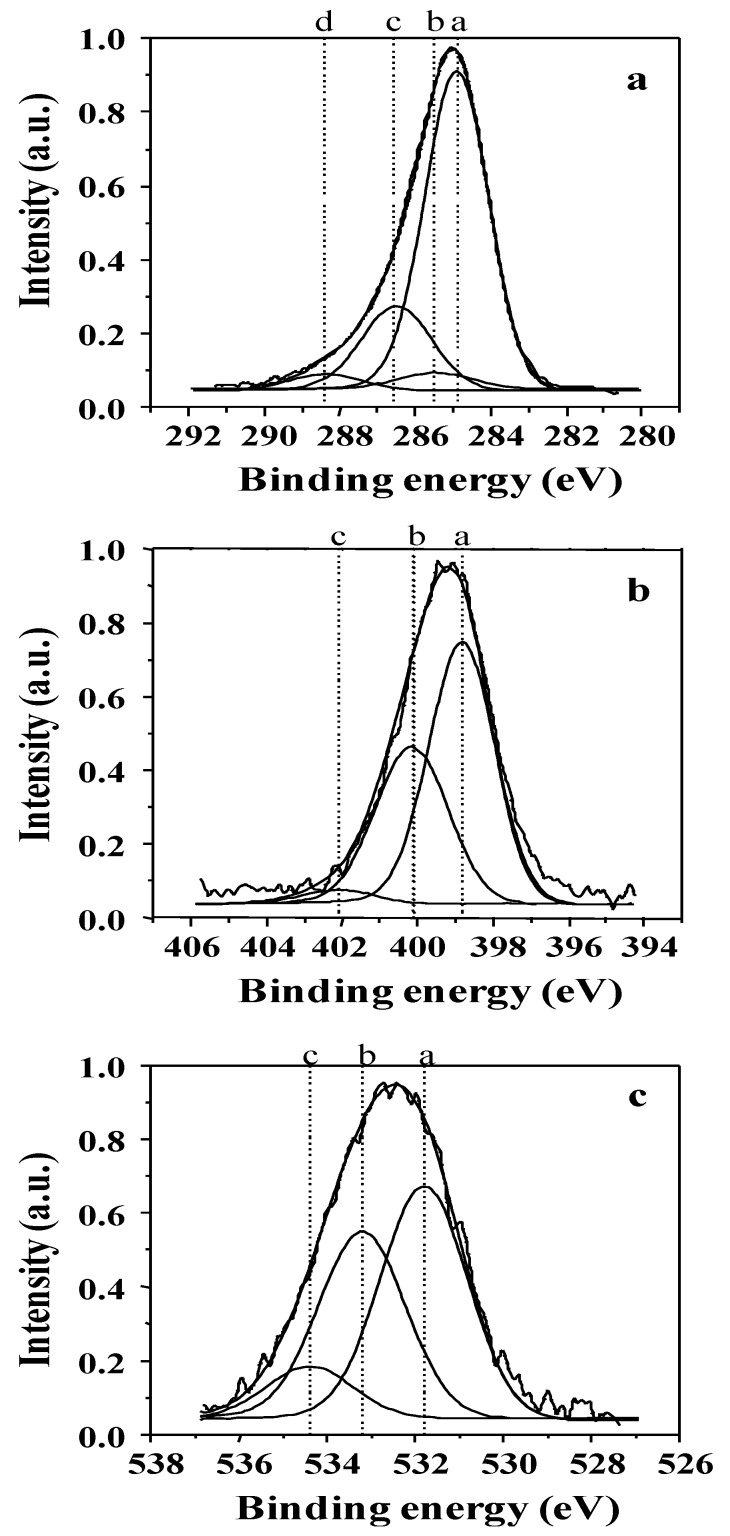
Examples of X-ray photoelectron spectroscopy (XPS) peak decomposition for (**a**) C_1s_; (**b**) N_1s_; and (**c**) O_1s_ photoelectrons in bulk of non-quaternized films analyzed with the SIA 200 instrument. The assignment of the various components is given in Table 1.

Chemical structure analysis of plasma deposits was performed using the XPS technique and correlated with plasma operating parameters. Typical decompositions of the C_1s_, N_1s_ and O_1s_ photoelectron peaks for non-quaternized film bulk are shown in Figure 5 and the corresponding bond energy and assignments are given in Table 1. Comparison between XPS composition in the bulk and at the film surface (not shown here) shows that the bulk composition is 5 at % less enriched in oxygen than the film surface, which is due to a pollution of the latter by atmospheric oxygen once returning the samples to room environment. The presence of oxygen in the bulk (whereas no oxygen is present in the precursor) comes from the residual oxygen contained in the plasma chamber, which is difficult to remove completely even after long vacuum pumping times. 

**Table 1:** Assignment of the XPS photoelectron peak decomposition for C_1s_, N_1s_ and O_1s_ photoelectrons.

Photoelectron	Peak	Bonding energy (eV)	FWHM (eV)	Assignment	References
C_1s_	a	284.8 ± 0.1	2.1	C–C(–C), C–H	[32,33,34]
	b	285.5 ± 0.1	2.2	sp^2^ C=N, C–C(–O), C–C(–N)	[35,36]
	c	286.6 ± 0.1	2.2	C–N, C–O (OCN)	[33,35]
	d	288.4 ± 0.1	2.3	C=O	[32,35,36]
N_1s_	a	398.6 ± 0.1	2.2	sp^3^ N–C	[37,38,39]
	b	400.1 ± 0.1	2.2	sp^2^ N=C	[40]
	c	402.1 ± 0.1	2.3	N–N, N–O	[39,41,42]
O_1s_	a	531.8 ± 0.1	2.2	O=C	[32,43]
	b	533.2 ± 0.1	2.2	O–C	[35,36]
	c	534.5 ± 0.1	2.3	O–H	[32,43]

**Figure 6:**
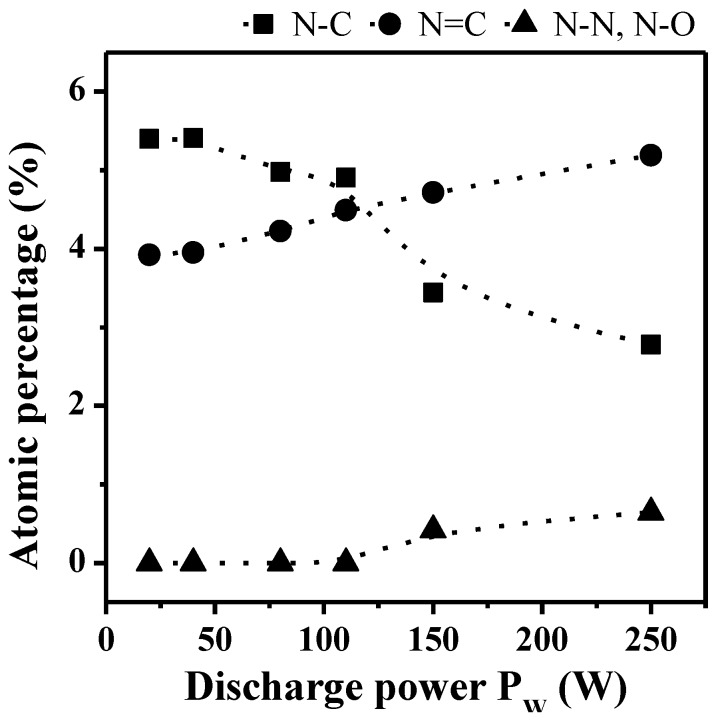
Evolution of the bonds proportion for the N_1s_ photoelectron peaks in the bulk of non-quaternized films (analyzed with the SIA 200 instrument) as a function of the discharge power *P_w_* (*DC* = 100%).

Decomposition of the N_1s_ photoelectron peak exhibits three components at *ca.* 398.6, 400.1 and 402.1 eV assigned to sp^3^ N–C; sp^2^ N=C and N–N or N–O bonds respectively (Figure 5b and Table 1). The evolution of the different nitrogen chemical bonds in the bulk as a function of the discharge power and for a *DC* of 100% is given in Figure 6. The fraction of sp^3^ N–C bonds, correlated to the presence of primary, secondary and tertiary amine functions in the films, is prominent at lower *P_w_* values and decreases progressively with increasing *P_w_* to become lower than the fraction of sp^2^ N=C bonds, the latter increasing steadily with *P_w_* increase. This phenomenon is correlated to the presence of less fragmented species for lower *P_w_* values, in good agreement with the CAP process (meaning that at lower power values the structure of the monomer, containing sp^3^ C–N bonds, is more retained than at higher power values, at which fragmentation is more important). At very high *P_w_* values, N–N or N–O bonds are appearing in small proportions, here again characteristic of the species fractioning.

**Figure 7:**
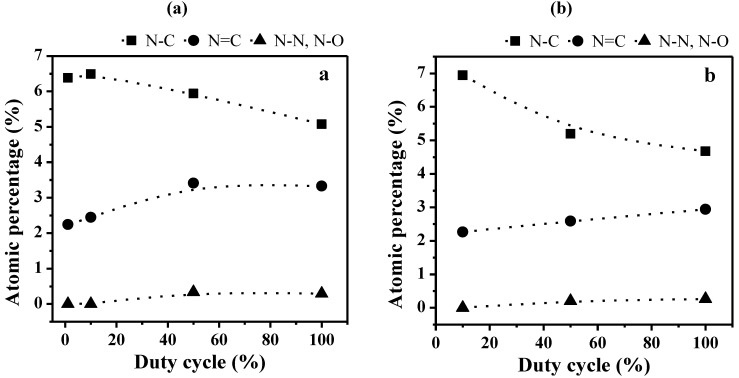
Evolution of the bonds proportion for the N_1s_ photoelectron peaks in the bulk of non-quaternized films (analyzed with the SIA 200 instrument) as a function of the duty cycle (*DC*) for plasma polymers prepared at (**a**) 40 W and (**b**) 150 W.

Similar behaviors are observed in the case of two sets of plasma films prepared at *P_w_* values of 40 and 150 W and increasing *DC* (Figure 7). Indeed, the proportion of sp^3^ N–C bonds is decreasing with the *DC* increase, together with increase of the sp^2^ N=C and N–N or N–O bonds. Since the average input power *P_A_* is increasing with the *DC* value, the evolution of the different nitrogen bonds is here again related to the presence of less fragmented molecular units in the polymer matrix for soft plasma conditions, *i.e.*, lower *P_A_* and *DC* values, and to more fragmented units for harder plasma conditions, *i.e.*, higher *P_A_* and *DC* values. The correspondence with the CAP process is even confirmed by the more significant decrease of the proportion of sp^3^ N–C bonds with increasing *P_w_* from 40 to 150 W (comparing Figure 7a and Figure 7b).

**Figure 8:**
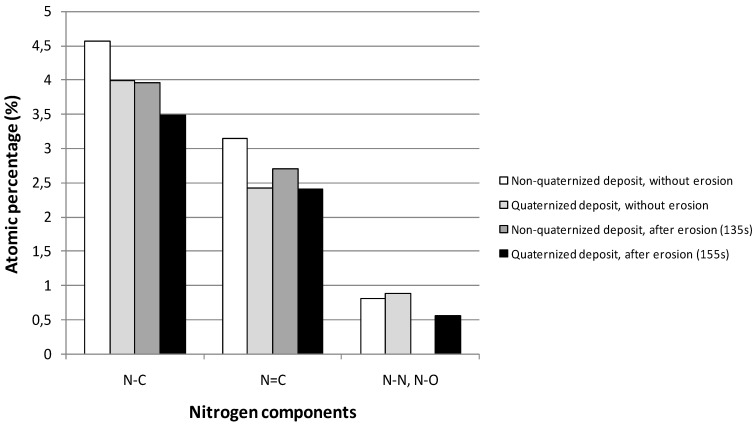
Evolution of the different components of the N_1s_ peak before and after quaternization, both at the surface (without erosion) and in the bulk (after erosion). Plasma deposition was performed in the following conditions: *P_w_* = 40 W, *DC* = 10% and *τ* = 10 min. XPS analyses were performed on the ESCALAB 250 instrument.

XPS measurements were performed on quaternized samples in order to depict the effect of the quaternization on the structure of plasma deposits. Figure 8 shows the atomic percentages of N_1s_ components for a film deposited in the following conditions: *P_w_* = 40 W, *DC* = 10% and *τ* = 10 min, before and after quaternization, both at the surface (without erosion) and in the bulk (after erosion). No significant evolution of the N_1s_ components can be depicted (it would be the same observation for C_1s_ components and whatever the plasma conditions for the preparation of films); so the efficiency of the quaternization reaction (replacement of N–H bonds by N–C bonds in amine functions) has not been proved. 

### 2.2. Transport Properties

#### 2.2.1. Water Content, *IEC* and Hydroxide Conductivity

In the case of argon plasma treatment, only plasma conditions using *DC* = 100%, *τ* = 10 min and varying *P_w_* values in the range 40–90 W are concerned for water content, *IEC* and conductivity measurements. The water content of plasma modified ADP-Morgane^®^ membranes is comprised in the range 26–37.5 wt % depending on *P_w_*. These values are above the value of pristine ADP-Morgane^®^ membrane (22 wt %) showing that the modified membranes have a higher capability of adsorbing water, directly related to the hydrophilization inherent to plasma treatment as previously mentioned. Concerning the AMELI-32^®^ membranes, the water content values are contained in the range 270 to 340 wt % depending on *P_w_*. The water content of some plasma modified samples is above the value of pristine AMELI-32^®^ membrane (304 wt %) and the one of others is below. However, one can notice the very high hydrophilic character of such AMELI-32^®^ membranes, whether modified or not, which is certainly a big asset for fuel cell application. The *IEC* of plasma treated ADP-Morgane^®^ membranes is decreasing with *P_w_* increase, from 2.3 to 1.85 mequiv g^−1^, and is above the unmodified ADP-Morgane^®^ one (1.8 mequiv g^−1^). The *IEC* decrease with *P_w_* increase may be associated to a rising ablation of functional groups responsible for the ion exchange, *i.e.*, quaternary ammonium groups, and/or to an enhanced cross-linking at the membranes surface, reducing the accessibility to the functional groups [27,30,31]. Additionally, the improved *IEC* of the plasma treated membranes compared to the pristine ADP-Morgane^®^ membrane might be connected to the hydrophilization of the membrane surface (inherent to plasma treatment) enhancing the water accessibility and consequently anions accessibility to the exchangeable sites. The *IEC* values of the AMELI-32^®^ membranes are contained in the range 1.86 to 2.89 mequiv g^−1^ depending on *P_w_*. These values are below the one of the pristine AMELI-32^®^ membrane, and this might be explained either by the ablation of functional groups and/or the enhanced cross-linking of the membranes surface [27,30,31]. The anionic conductivity of plasma treated ADP-Morgane^®^ membranes is in the range 82–187 mS cm^−1^ depending on *P_w_*. These values are above the value of untreated ADP-Morgane^®^ (51 mS cm^−1^) but are below the one of Nafion^®^, 194 mS cm^−1^ (as a reference membrane characterized in acidic medium in the same measurement cell). The conductivity values of the treated AMELI-32^®^ membranes (between 8.4 and 11.3 mS cm^−1^) are higher than the one of the pristine AMELI-32^®^ membrane (7.5 mS cm^−1^) but lower than those of the treated or untreated ADP-Morgane^®^ membranes. 

**Figure 9:**
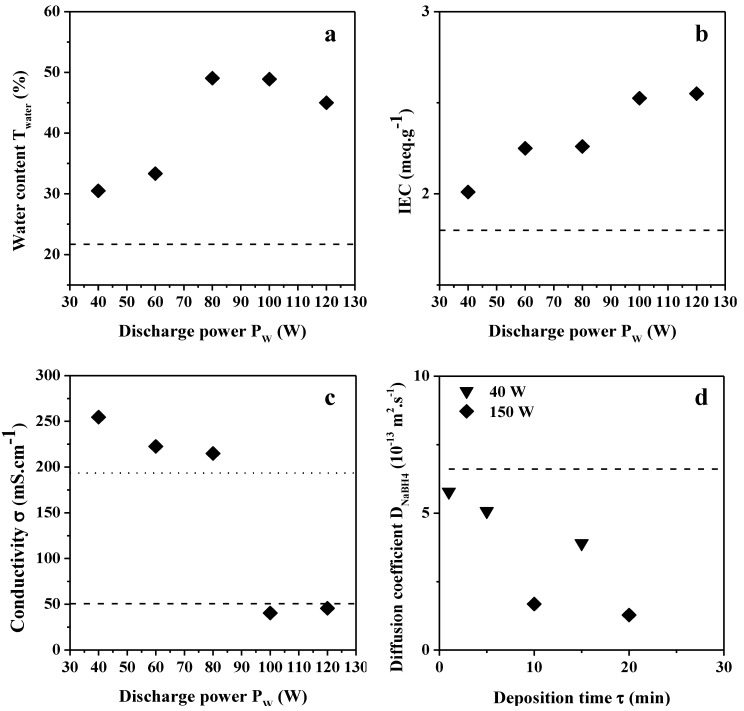
Evolution of the (**a**) water content; (**b**) ion exchange capacity and (**c**) ionic conductivity of modified ADP-Morgane^®^ membranes as a function of the power discharge used in the preparation of plasma films (*DC* = 10%, *τ* = 10 min); (**d**) Evolution of the NaBH_4_ diffusion coefficient of modified ADP-Morgane^®^ membranes as a function of the deposition time of plasma films (*P_w_* = 40 or 150 W, *DC* = 10%). Dashed and dotted lines correspond to the reference properties of unmodified ADP-Morgane^®^ and Nafion^®^ membranes, respectively.

In the case of plasma deposition, only plasma conditions using the lowest *DC* value (10%), *τ* = 10 min and varying *P_w_* values in the range 40–120 W are concerned for water content, *IEC* and conductivity measurements. Indeed, one can expect better anion transport properties for films synthesized at low *DC* for which the proportion of sp^3^ N–C environments is higher than in films prepared at high *DC* (as demonstrated by XPS analysis). The water content of the modified membranes is comprised in the range 30–35 wt % for *P_w_* values below 70 W and in the range 45–50 wt % for values above, which is also much higher than the unmodified ADP-Morgane^®^ membrane (Figure 9a). The *IEC* of the modified membranes is increasing from 2.0 to 2.5 mequiv g^−1^ with *P_w_* increase (Figure 9b). Here again, one can observe an improvement of the *IEC* compared to the pristine ADP-Morgane^®^ membrane (1.8 mequiv g^−1^). One can suppose that, when the plasma discharge has just been initiated, the surface of the ADP-Morgane^®^ membrane is slightly etched before the plasma film is depositing (as predicted by the CAP process [27,30,31]). Now, it is possible that plasma etching of the ADP-Morgane^®^ membrane enables the removal of a non-ionic conductive contaminated upper layer (issued from preparation procedure of the commercial membrane), inducing higher water content and *IEC*. Moreover, the deposited plasma film is quite highly oxidized at the surface (as previously shown by XPS) which can also contribute to hydrophilic enhancement and so to higher water content and *IEC*. Both reasons explain the improvement of the water content and *IEC* for plasma-deposited ADP-Morgane^®^ membranes in comparison with unmodified one. Very logically, these etching and oxidation phenomena are more pronounced at high *P_w_* values than at low ones, which explains the increase of the water content and *IEC* with *P_w_*. The anionic conductivity of the modified membranes is in the range 200–250 mS cm^−1^ for *P_w_* values below 90 W and around 50 mS cm^−1^ for *P_w_* values above (Figure 9c). These conductivities are globally higher than the pristine ADP-Morgane^®^ membrane one (51 mS cm^−1^). 

The improvement in term of conductivity issued from the plasma deposition can be explained by the etching/oxidation effects previously mentioned. Nevertheless, by supposing that these effects are the only ones controlling the conductivity, one would expect that if the water content and *IEC* are increasing with *P_w_*, since water molecules play the role of carriers and *IEC* is correlated to the number of cationic sites, conductivity would increase as well; though the opposite behavior is happening. This discrepancy proves that the etching/oxidation effects (which should lead to the conductivity increase with *P_w_*) are not the only phenomena controlling the conductivity. 

In fact, the conductivity is also highly controlled by the plasma film density. At low *P_w_* values, plasma deposits are composed of less fragmented species, allowing more space and higher anions diffusion within the matrix; whereas at high *P_w_* values, plasma films contain more fragmented species, are more cross-linked and denser, and consequently are less conductive. To our knowledge, the influence of the CAP process (competition between ablation and deposition) on transport properties of plasma modified anionic membranes has not been evidenced as clearly before as here [26,27,29,30,31,18,44]. In addition, the influence of quaternization on water content, *IEC* and conductivity has been investigated for two different samples. Quaternization seems not to affect significantly the water content and *IEC* for both investigated samples. However, it has been shown that quaternization has a beneficial effect on conductivity for membranes modified in soft plasma conditions, e.g., *P_w_* = 40 W, *DC* = 10% and *τ* = 15 or 20 min, for which an improvement of up to 150% may be observed. On the opposite, quaternization performed on membranes modified in harder plasma conditions, e.g., *P_w_* = 80 W, *DC* = 10% and *τ* = 10 min, leads to a reduction of 30% of the conductivity. This reduction of conductivity for the most highly cross-linked films is certainly due to a prohibitive free volume decrease in the film matrix directly related to the addition of methyl groups issued from the quaternization. 

#### 2.2.2. NaBH4 and Glycerol Permeability

NaBH_4_ diffusion coefficients were measured for a series of argon plasma treated ADP-Morgane^®^ membranes. The modification seems to have an unpredictable effect since the diffusion coefficient values are either reduced to 5 × 10^−13^ or increased to 13.2 × 10^−13^ m^2^ s^−1^ (pristine ADP-Morgane^®^ 6.6 × 10^−13^ m^2^ s^−1^), without any obvious influence of *P_w_*. Concerning the glycerol diffusion coefficient for the same series of membranes, it seems that the plasma modification reduces it from 6.9 × 10^−10^ m^2^ s^−1^ for the pristine ADP-Morgane^®^ membrane to 6 × 10^−10^ m^2^ s^−1^ for the plasma modified ones. Regarding the AMELI-32^®^ membranes, the glycerol diffusion coefficient is reduced from 3.5 × 10^−9^ m^2^ s^−1^ for the pristine membrane to 1.1 × 10^−9^ m^2^ s^−1^ for the only plasma modified one (*P_w_* = 60 W, *τ* = 10 min). This significant reduction of glycerol permeability is probably due to an increased cross-linking of the membranes surface [27,30,31], and shows the interesting potential of plasma treated ADP-Morgane^®^ and AMELI-32^®^ membranes for applications as solid electrolytes.

NaBH_4_ diffusion coefficients were also measured for two sets of modified ADP-Morgane^®^ membranes on which TAA thin plasma films were deposited at *P_w_* = 40 or 150 W (*DC* = 10%) and for different *τ* values (Figure 9d). Logically, the NaBH_4 _diffusion coefficients of the modified membranes are lower than the one of pristine ADP-Morgane^®^ membrane. Moreover, the NaBH_4 _diffusion coefficients decrease with *τ* and *P_w_* increase, due to film thickness increase and film densification, respectively. Unfortunately, quaternization induces an increase of the diffusion coefficients by factors of 2 to 3 in the range 1 × 10^−13^–2 × 10^−12^ m^2^ s^−1^. Glycerol diffusion coefficients were also measured on two pieces of plasma deposited ADP-Morgane^®^ (TAA deposit at *P_w_* = 80 W, *DC* = 10% and *τ* = 10 min), one of them being quaternized. The diffusion coefficient values are 4.4 × 10^−10^ and 4.1 × 10^−10^ m^2^ s^−1^ for TAA polymer deposited ADP-Morgane^®^ and quaternized TAA polymer deposited ADP-Morgane^®^, respectively. Thus, glycerol permeability is slightly reduced by the deposition of a TAA polymer film on the membrane, though quaternization seems not to affect much this property.

### 2.3. Fuel Cell Tests

**Figure 10:**
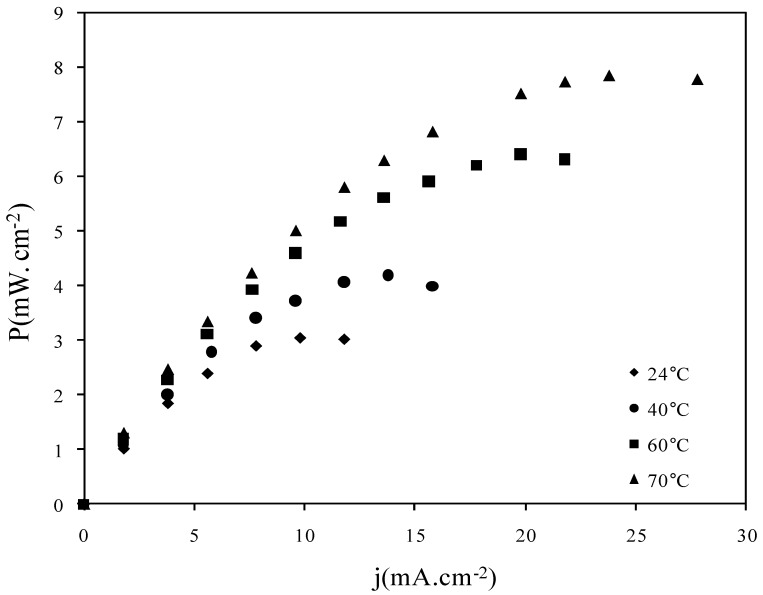
Power density curves recorded at different temperatures in SAFC fitted with MEA realized with pristine ADP-Morgane^®^ membrane.

Fuel cell tests only concern ADP-Morgane^®^ membrane. The increase of the SAFC working temperature is one of the major key for the development of this technology. So power density curves were recorded at different temperatures using ADP-Morgane^®^ non-treated membrane (Figure 10). The temperature increase from 24 °C to 70 °C leads to the enhancement of the maximal power density (from 3 mW cm^−2^ at 24 °C up to 7.8 mW cm^−2^ at 70 °C); the effect seems to be related to the improvement of reaction kinetics at the electrodes. 

**Figure 11:**
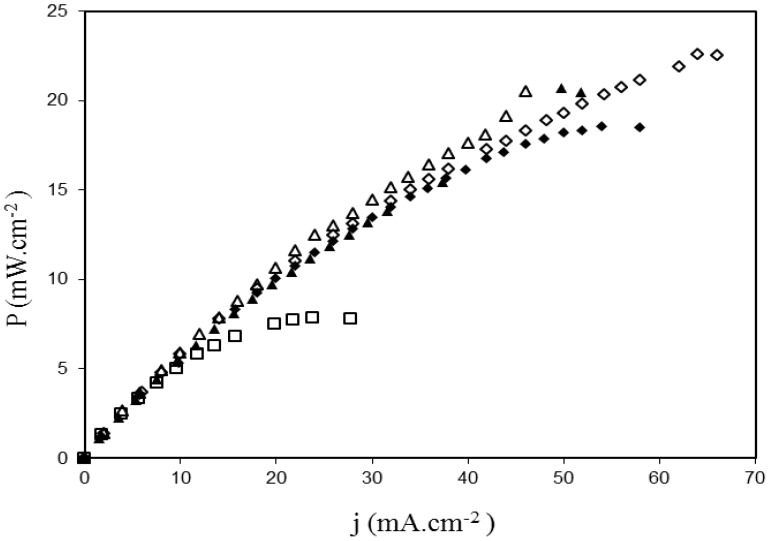
Power density curves recorded in SAFCs at 80°C fitted with MEA realized with ADP-Morgane^®^ membranes. Membrane □ is pristine ADP-Morgane^®^. Membranes ♦ and ▲ are one-side treated membranes at 2 and 80 W (10 min), respectively (the treated side is positioned close to the anode during fuel cell tests). Membranes ◊ and Δ are both-sides treated membranes at 2 and 80 W (10 min), respectively.

Figure 11 demonstrates that the use of argon plasma treated membranes (2 or 80 W, 10 min) results in the improvement of the cell electrical performance. Indeed, the comparison of results as a function of the plasma input power shows that, whether the membrane was modified on one or both side(s), the best performance is obtained with the lowest value of the plasma input power (*i.e.*, 2 W). For the best plasma input power of 2 W, the plasma treatment on both sides of the membrane is more efficient (22.6 mW cm^−2^) than the treatment on only one side (18.5 mW cm^−2^). No significant difference has been detected between one and both side(s) treatment for the plasma input power of 80 W. When compared to the pristine ADP-Morgane^®^ membrane (7.8 mW cm^−2^ at 70 °C), the maximum power density delivered by the best argon plasma treated membrane (2 W, 10 min, treatment on both sides of membrane) is almost three times higher (22.6 mW cm^−2^ at 80 °C). The SAFC test of the best non-quaternized plasma deposited ADP-Morgane^®^ membrane (TAA deposit at *P_w_* = 40 W, *DC* = 10% and *τ* = 20 min) also revealed better performance than the untreated membrane (maximum power density equal to 21 mW cm^−2^ at 80 °C). These noticeable improvements are consistent with the conductivity, water content, *IEC* and glycerol retention enhancements previously mentioned.

**Figure 12:**
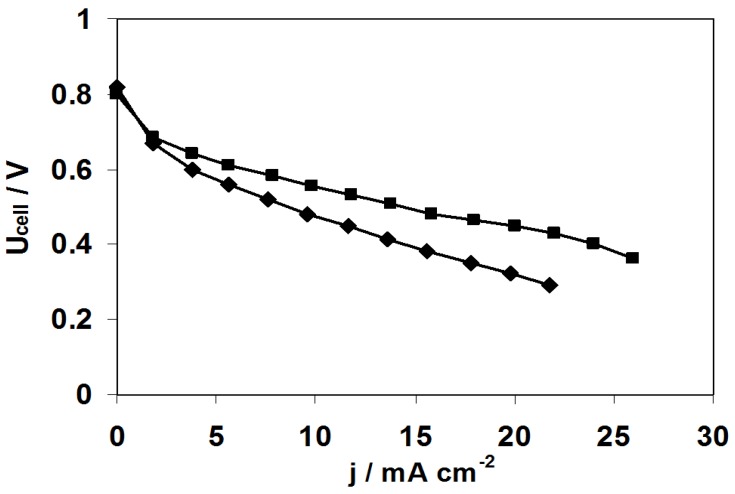
Polarisation curves recorded at 60 °C in SAFC fitted with MEA realized with pristine ADP-Morgane^®^ membrane (◆) and with one-side argon plasma treated membrane at 2 W for 10 min (■). *P_fuel_* = *P_O2_* = 1 atm, fuel flow rate = 0.8 mL min^−1^, O_2_ flow rate = 40 mL min^−1^.

In order to better understand the effect of the plasma treatment on the enhancement of the fuel cell performance, polarization curves have also been registered. Figure 12 illustrates the case of the one-side argon plasma treatment (2 W, 10 min) which is representative of all other plasma treatments. It is shown that there is no effect of the plasma treatment in the activation region (low current density and high cell voltage region), whereas there is one in the ohmic region (linear part of the polarization curves), which is certainly related not only to the difference in membrane conductivity between untreated and treated membranes but also to the difference in membrane-electrode interface conductivity. Indeed, the effect of membrane surface etching inherent to plasma treatment may have increased both the adherence of the membrane on the electrode and the surface area contact between the membrane and the electrode, favoring the hydroxyl ions transfer and mimicking mass transfer enhancement at high current densities.

## 3. Experimental Section

### 3.1. Plasma Process Equipment

**Figure 13:**
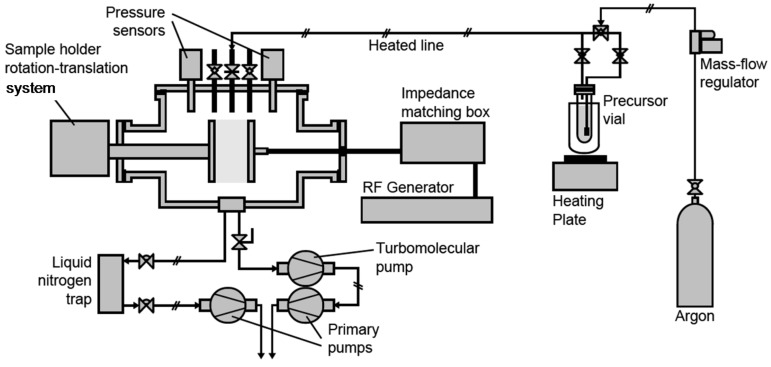
Plasma process equipment.

Plasma process was performed in a 30 L commercial capacitively coupled plasma reactor built by MECA2000 (depicted in Figure 13), pumped through a turbomolecular pump (Adixen Pascal 2015 SD coupled with a Boc Edwards EXC 120 module) for pre-treatment high vacuum and a primary pump (Adixen Pascal 2015 SD) for the plasma treatment or deposition. 

A liquid nitrogen trap was positioned between the plasma chamber and the primary pump in order to trap the precursor and fragments vapors in the case of plasma deposition. The pressure inside the chamber was sensed and monitored respectively by a Leybold Vakuum Ceravak gauge and Edwards Speedivalve valve. A 13.56 MHz radiofrequency (RF) source (Dressler CESAR 136) was used to supply power to the parallel and vertical plate electrodes (gap between both electrodes: 2 cm). The RF and grounded electrodes were disk electrodes (10 cm diameter), being respectively immovable and rotative (speed ~6 rpm). Argon (Air liquid, purity > 99.999%), used as main gaseous phase for plasma treatment or as carrier gas for plasma deposition, was fed into the system via a UNIT Instruments Inc. (UFC1000) gas flow meter. For plasma deposition, the precursor triallylamine (Aldrich, 99%), named TAA in this paper, was contained in a flask maintained at a temperature of 45 °C. The stainless steel pipe between the precursor flask and the reactor was maintained at a temperature of 60 °C, to avoid precursor condensation in the pipe. 

### 3.2. Plasma Process Procedure

This section describes a standard procedure for plasma treatment using argon as gaseous phase (on both ADP-Morgane^®^ and AMELI-32^®^) and plasma deposition using triallylamine as precursor (on ADP-Morgane^®^ only). First, a piece of membrane (from 20 × 25 up to 30 × 30 mm^2^) and a silicon wafer (from 10 × 20 up to 30 × 30 mm^2^) were fixed, using Kapton tape, on the removable holder, which was then positioned on the anode port. The plasma chamber was then evacuated using the primary vacuum system until approaching the limit pressure (P < 10^−2^ mbar) and further evacuated for at least one hour at this pressure. The rotation of the anode was started, before switching to the secondary vacuum system. In the case of plasma deposition, the liquid nitrogen trap was filled and its level regularly adjusted for all the process long. The chamber was then evacuated until reaching a pressure of *ca.* 10^−5^ mbar and the vacuum was maintained at this pressure for at least 15 min. The system was then switched back to the primary vacuum setup and the argon flow started and set to a mass flow opening of 30% or 1.7%, for plasma treatment or deposition respectively. Once the internal pressure of argon in the reactor reached equilibrium, the plasma glow discharge was switched on and maintained for the desired duration in the case of plasma treatment. In the case of plasma deposition, the injection pipe system was flipped to the argon-TAA mixture; once an equilibrium pressure of *ca.* 0.9–1.3 × 10^−1^ mbar was reached, the plasma glow discharge was switched on and maintained for the desired duration. After the plasma discharge was switched off, the argon stream was set to a mass flow of 50% and maintained for half an hour (after flipping back of the injection pipe system to the sole argon line in the case of plasma deposition). After stopping the argon flow, the evacuation was further continued using the primary vacuum system for at least 30 min, before returning the chamber to atmospheric pressure and recovering the samples. 

The parametric study consisted in varying the discharge power (*P_w_*) from 2 to 100 W (for argon plasma treatment) or from 20 to 250 W (for plasma deposition); the duty cycle (*DC*, defined as the ratio of the effective on light plasma duration to the overall plasma process duration) was equal to 10% or 100% and the plasma glow duration (*τ*) was fixed at 10 or 20 min (for argon plasma treatment) or varied from 2 to 60 min (for plasma deposition). The average input power, defined as *P_w_* × *DC*, is noted *P_A_*. 

In the case of argon plasma treatment, membranes were treated on one or both side(s); in the latter case, the plasma treatment procedure was implemented twice, reversing the membrane on the substrate holder between both procedures. In the case of plasma deposition, some films were post-treated by methyliodide (at 85 °C for 16 h in a 10:90 v/v methyliodide/acetonitrile mixture) for the quaternization of the amine functions.

### 3.3. Membranes Characterization

SEM measurements were conducted on a Hitachi S4500 instrument under an excitation voltage value between 2 and 8 kV depending on each sample’s surface charging. Before analysis, pieces of membranes were immersed in liquid nitrogen and broken in order to have a neat cut of the membranes’ cross-section; then pieces of membrane were stuck on double face tape on microscope support and Pt-metalized by sputtering under vacuum.

XPS analyses were conducted to depict the chemical composition of plasma films (deposited on silicon wafer). They were performed on an SIA 200 instrument from Riber Cameca UHV or on an ESCALAB 250 instrument from Thermo Electron. The source of excitation was the Al K*_α_* X-ray at 1486.6 eV. The photoelectron spectra were calibrated in bond energy to the C–C/C–H bonds component of the carbon C_1s_ peak at 284.8 eV. Samples were analyzed both at their surface and in the bulk. The bulk was characterized after erosion of the deposits (using an argon ionic beam) for etching durations specifically adjusted according to, on the one hand, film thickness and hardness, and on the other hand, apparatus and energy of the argon ionic beam. Optimized etching durations were 600 s in the case of non-quaternized films bombarded with a 0.6 kV argon ionic beam on the SIA 200 instrument; they were 135 s and 155 s for non-quaternized and quaternized films, respectively, bombarded with a 2 kV argon ionic beam on the ESCALAB 250 instrument. 

*IEC* measurements were performed using a Tacussel TT-2 auto-titrator coupled to a Radiometer ABU901 auto-burette. Titration was based on the dosage of Cl^−^ anions delivered from a Cl^−^-membrane exchanged in a nitric acid solution. Typically, a piece of 1 cm^2^ of membrane was immersed in *ca.* 25 mL of HCl 1 mol L^−1^ under stirring during 1 h. The process was repeated once before recovering the piece of membrane, which was then copiously rinsed with ultrapure water (R ≤ 18.2 MΩ cm) before being immersed in a 50 mL solution of 1 mol L^−1^ HNO_3_ (volume precisely determined) for at least 12 h. At the end of this anion-exchange period, the piece of membrane was recovered, copiously rinsed with ultrapure water and retained for further analysis of the water content. The nitric solution was then titrated using a 10^−3^ mol L^−1^ silver nitrate solution and the Cl^−^ anion concentration of the anionic-exchange nitric acid solution determined (*C_Cl_*). The *IEC* value was calculated using the following formula: *IEC* = (*C_Cl_* × *V_HNO3_*)/*m_d_*, where *V_HNO3_* is the volume of the anionic-exchange solution (50 mL) and *m_d_* is the weight of the dried piece of membrane (determined during the water content analysis). Titration was performed at least three times on each anionic-exchange nitric acid solution.

Water content of the membranes was determined using a Mettler Toledo HR53 moisture analyzer. Typically, the 1 cm^2^ piece of membrane retained during the *IEC* determination was immersed in 25 mL ultrapure water for 1 h, then recovered and immersed in *ca.* 25 mL of HCl 0.1 mol L^−1^ solution for 2 h. The membrane was recovered; its faces quickly dried out with blotting paper, placed on the moisture analyzer’s balance and the heating process started to reach 120 °C. The measurement stopped when the weight on the balance was changing twice for less than 10^−3^ mg. The final weight of the membrane was considered as the one of the dried membrane *m_d_*. The weight measured right before starting the heating process was considered as the wet weight *m_w_*. The water content *T_water_* was calculated as follows: *T_water_* = 100 × (*m_w_* − *m_d_*)/*m_d_*.

EIS measurements were obtained at room temperature from alternative current impedance analysis using a Solartron 1260 impedance analyzer coupled to a Solartron 1287 potensiostat, over the frequency range 1–10^7^ Hz with controlled voltage. The home-made mercury cell used for the measurements is constituted by two identical compartments, carved in Teflon pieces, between which the membrane is blocked using Viton o-rings [45]. Prior to measurement, the membrane was immersed in a 1 mol L^−1^ NaOH solution for 24 h and afterwards rinsed and soaked in ultrapure water for 24 h. Right before EIS measurement, the membrane was removed from water (its faces wiped out with blotting paper, its wet thickness measured using a micrometer) and was finally placed in the mercury cell. All the process shall be carried out as swiftly as possible, usually in less than 2 min. Once the membrane was positioned in the cell, the two compartments were filled with mercury; a Pt electrode was dipped in each one and connected to the analyzer. The resistance *R* of the membrane was derived from the intersection of the high-frequency semicircle on a complex impedance plane with the real *Re(Z)* axis. The conductivity was calculated using the relationship *σ* = *Th_w_*/(*R* × *S*), where *Th_w_* is the wet thickness of the membrane, S is the active surface of measurement (0.785 cm^2^ here).

NaBH_4_ and glycerol permeability were measured using a Hittorf diffusion cell constituted by two carved Teflon compartments compressing the membrane. One compartment contained 20 mL of ultrapure water and the other 200 mL of either a solution composed of a mixture of NaOH (1 mol L^−1^) and NaBH_4_ (2 mol L^−1^) or a solution of glycerol (2.5 mol L^−1^). Samples of the smallest compartment solution were taken at regular intervals and analyzed for the amount of NaBH_4_ or glycerol present. Boron elemental analysis was carried out by the Service Central d’Analyse of the CNRS (Vernaison, France) and glycerol was titrated by using FT-IR. Measurements were performed in attenuated total reflectance (ATR) mode on either a Nicolet 710 Thermo Nicolet instrument or a Nexus Thermo Electron instrument. Spectra were recorded in transmittance mode between 400 and 4,000 cm^−1^ wavenumbers, with a resolution of 4 cm^−1^ and by accumulating 32 scans. Glycerol content was determined by integration of the bands observed in the range 950–1150 cm^−1^, characteristic of the C–O stretching of primary and secondary alcohols [11,46]. Calibration of the spectrometer was performed using 0.5, 1.0, 2.0 and 2.5 mol L^−1^ glycerol solutions. Diffusion coefficients were calculated, for a given time of permeation, from the slope α of the Δ*n* = F(Δ*C*) curves, where Δ*n* represents the molar amount of permeate which has actually permeated and Δ*C* the permeate concentration gradient between the up- and downstream compartments. The diffusion coefficient value *D* was calculated from the formula *D* = *α* × *Th*/(Δ*t* × *S*), where *Th* is the initial thickness of the membrane, Δ*t* the time of permeation and *S* the active surface of measurement (0.785 or 3.14 cm^2^ here, depending on the permeation cell used). The flow of permeate *J* was calculated from the following formula *J* = (*D* × Δ*C_0_*)/Th, where Δ*C_0_* is the initial permeate concentration gradient.

### 3.4. Membrane-Electrodes Assemblies Preparation and Fuel Cell Tests

Virgin ADP-Morgan^®^ and some modified membranes of the same kind were tested in fuel cells. Before integration in membrane-electrodes assemblies (MEA), membranes were activated by immersion in a freshly prepared solution of 1M NaOH for 24 h at room temperature, and then rinsed with ultrapure water and hydrated for one hour. The manufacturing of MEA was performed (at P’ Institute—UPR CNRS 3346) by mechanical pressing of two electrodes against a membrane at ambient temperature. Electrodes were prepared (at LACCO laboratory—UMR CNRS 6503) by deposition of a catalyst ink (Pt (40 wt %)/C, 2 mg_Pt_ cm^−2^) on a home-made gas diffusion layer [12]. The fuel cell tests were performed (at P’ Institute—UPR CNRS 3346) using a test bench of Fuel Cell Technologies, Inc. including a single cell with an active area of 5 cm^2^. The MEA was placed between two mono-polar plates and assembled under 8 Nm torque wrench [47,48,49,50]. The fuel cell ran with pure oxygen as oxidant at the cathode (100 mL/min, 1 atm. back pressure, 20–80 °C) and a glycerol 1M/NaOH 4M solution as fuel at the anode (10 mL/min, 1 atm. back pressure, 20 °C).

## 4. Conclusions

In this work, commercial ADP-Morgane^®^ fluorinated polymer membranes and a new brand of cross-linked poly(aryl-ether) polymer membranes, named AMELI-32^®^, both containing quaternary ammonium functionalities, have been modified by argon plasma treatment or plasma assisted deposition of thin films using triallylamine as precursor. SEM analysis has shown that etching/cross-linking occurs for both membranes under argon plasma treatment, highlighting reduced grain size of the polymer matrix or lower mechanical properties in the case of the ADP-Morgane^®^ membrane. In the case of plasma deposition, the growth rate of the thin films presents a bimodal evolution as a function of the average power input P_A_ (on both sides of 40 W) characteristic of the competitive ablation and polymerization process (CAP process) differentiating two different plasma regimes: an energy-deficient one (under soft plasma conditions, here P_A_ < 40 W) leading to soft, less fragmented polymers, where the structure of the monomer is retained, and an energy-surplus one (under hard plasma conditions, here P_A_ > 40 W) leading to highly fragmented and more dense polymers. The XPS characterization of non-quaternized plasma films has shown that sp^3^ N–C bond population decreases in the plasma film network, while the sp^2^ N=C bond population increases, with the hardness of plasma conditions, *i.e.*, for increased values of input power (*P_w_*) and/or duty cycle (*DC*). Efficiency of the quaternization process could not be proved by XPS analysis. 

In the case of argon plasma treatment, the improved *IEC*, water uptake and hydroxide conductivity of the plasma treated membranes compared to the pristine ones might be attributed to the hydrophilization of the membrane surface (inherent to plasma treatment) enhancing the water accessibility and consequently anions accessibility to the exchangeable sites. The significant reduction of glycerol permeability under the effect of plasma treatment is probably due to an increased cross-linking of the membranes surface. In the case of plasma deposited membranes, the study of functional properties has shown that water content and ionic exchange capacity are increasing with the discharge power *P_w_* increase, directly related to the etching/oxidation effects of the plasma process, while the hydroxide conductivity is decreasing under the major effect of film cross-linking. The NaBH_4_ and glycerol diffusion coefficients of modified Morgane-ADP^®^ membranes decrease with *P_w_* increase, which is also directly related to cross-linking effect. Independently of the plasma deposition conditions, water content, ionic exchange capacity, hydroxide conductivity and fuel retention of modified membranes are higher than those of pristine one. Quaternization of films has a beneficial effect on ionic conductivity for membranes modified in soft plasma conditions, but a detrimental one on the fuel retention.

As a consequence of functional properties improvements, the plasma modification of the commercial ADP-Morgane^®^ membrane allows to increase the maximum power density of alkaline MEA by a factor of three (up to 22.6 mW cm^−2^ at 80 °C).
